# Construction and characterization of a contagious ecthyma virus double-gene deletion strain and evaluation of its potential as a live-attenuated vaccine in goat

**DOI:** 10.3389/fimmu.2022.961287

**Published:** 2022-09-02

**Authors:** Zhen Zhu, Guanggang Qu, Jige Du, Changjiang Wang, Yong Chen, Zhanning Shen, Zhiyu Zhou, Chunsheng Yin, Xiaoyun Chen

**Affiliations:** ^1^ China Institute of Veterinary Drug Control, Beijing, China; ^2^ Shandong Binzhou Animal Science and Veterinary Medicine Academy, Binzhou, China

**Keywords:** ORFV, double gene deletion, *cbp*, *gif*, recombinant virus, safety, protection efficacy, vaccine

## Abstract

Contagious ecthyma is a highly contagious viral disease with zoonotic significance caused by orf virus (ORFV) that affects domestic, ruminants and humans. Live attenuated virus and attenuated tissue culture vaccines are widely used in the fight against ORFV, however, the conventional attenuated vaccine strains have many drawbacks. The aim of this project was to construct a promising contagious ecthyma vaccine strain with safety, high protection efficacy and accessibility by genetic manipulation to against the disease. Using a natural ORFV-GS14 strain as the parental virus, recombinant virus, rGS14-ΔCBP-ΔGIF, with double deletions in the genes encoding the chemokine binding protein (CBP) and granulocyte/macrophage colony-stimulating factor inhibitory factor (GIF) was generated and characterized *in vitro* and *in vivo*. Results showed that the growth kinetics curve of rGS14-ΔCBP-ΔGIF and parental virus was consistent, both reaching plateau phase at 48 h post infection, which indicated that the double deletion of *cbp* and *gif* genes had little impact on the replication properties of the recombinant virus in primary goat testis (PGT) cell cultures compared with the parental virus. The safety of the double gene-deleted virus was evaluated in lambs. The lambs were monitored for 21 days post infection of the recombinant virus and no ORFV associated symptoms were observed in 21 days post-infection except for slight fever and anorexia in 5 days post-infection, and all lambs inoculated with either recombinant virus or PBS exhibited no clinical signs. To assess the protection efficacy of the rGS14-ΔCBP-ΔGIF, groups of four lambs each were inoculated with rGS14-ΔCBP-ΔGIF, rGS14-ΔCBP, rGS14-ΔGIF or PBS and challenged by a wild type virulent ORFV strain that was isolated from proliferative scabby lesions tissues of infected goat at 21-day post-inoculation. During 14 days post-challenging, lambs inoculated with rGS14-ΔCBP-ΔGIF all remained healthy with unimmunized group all infected, while the single gene-deleted viruses only protected 40% to 50% animals. These results indicated that the double gene-deleted recombinant virus could provide complete protection against virulent ORFV challenging. In conclusion, the double gene-deleted recombinant virus strain, rGS14-ΔCBP-ΔGIF, would be a promising candidate vaccine strains with safety, high protection efficacy and availability.

## Introduction

Contagious ecthyma also known as orf, is a highly contagious and economically significant zoonotic viral skin disease in sheep and goats. It is characterized by proliferative skin and mucous membrane of the oral cavity, tongue, lips and ears which are developed sequentially in the form of papules, vesicles, pustules, scabs (1). Generally, orf is a mild and self-limiting disease with regression of lesions within 1-2 months. However, it can be severe, long-lasting and generalized occasionally, and mortality rate can be high due to the debilitating nature of the disease along with secondary pathogen infections, especially in younger animals (2–3). This disease has been reported communicable to humans from infected small ruminants (4) and is mandatory to report due to its significant impact in animal husbandry.

Orf is caused by the epitheliotropic orf virus (ORFV), a member of the genus *Parapoxvirus* from Poxviridae, which has a linear double-stranded DNA genome of approximately 138 kbs encoding 132 genes (5–6). Many studies on the whole gene sequencing and analysis of ORFV have been reported (7–8). The genetic organization of ORFV exhibits the general patter of poxviruses family including a highly conserved core region in the middle of the genome and variable terminal regions at both ends (9). The central region contains essential genes encoding proteins involved in viral replication and the genesis of the viral structure, while genes in the terminal regions encode proteins mainly implicated in host range, pathogenesis and virulence (10). The *B2L* gene of ORFV is one of the important genes, which was usually used as a target in polymerase chain reaction (PCR) method to detect ORFV and used in phylogenetic analysis because of its specificity and conservation in ORFV (11). *F1L* is another conservative gene and its products was used for sero-diagnosis of orf in sheep and goats (12). It is well documented that *vir* is an important virulence gene encoding two nonstructural proteins, which can create a favorable condition for virus replication and proliferation (13). Besides, several immunomodulatory proteins (IMPs) and their encoding genes, such as chemokine inhibitor protein (CBP; *ORFV112*) (14), granulocyte/macrophage colony-stimulating factor inhibitory factor (GIF; *ORFV117*) (15), and interleukin 10 homologue (vIL-10; *ORFV127*) (16–17) have been identified. ORFV IMP genes are located near the genome termini and modulate host-innate and pro-inflammatory responses to infection. CBP is the product of *ORFV112* that is located at the right end of the genome and is capable of disrupting chemokine gradients thereby inhibiting immune cells migrating to sites of infection (14). It was reported that *ORFV112* deletion resulted in the attenuation of ORFV NZ2 (18). GIF is expressed in the late stage of infection and is the only known viral or cellular protein that has the dual activity of inhibiting host GM-CSF and IL-2 (14, 19). The function of GIF, similar to CBP, is reported to inhibit the trafficking of monocytes into the skin to preventing inflammatory response (19). These IMPs above are important virulence factors that contribute to ORFV pathogenesis in the natural host.

Vaccination is the efficient and cost-effective method to prevent the ORFV infection. Conventional vaccines have been found to be ineffective for contagious ecthyma by an *in-vivo* assay, where inoculation with inactivated ORFV could not prevent re-infection of the host (20), but live virus and attenuated tissue culture vaccines are reported to provide adequate protection against ORFV (21). In veterinary medicine, attenuated tissue culture or scab-based vaccines that are not fully attenuated and produced in sheep are used (22). However, the use of live virus vaccine leads to the potential risk of diffusing ORFV and there are reports of the vaccine used for ORFV resulting in human infection (4). On the other hand, conventional attenuated virus vaccine based on virus passaged *in vivo* also has the risk of virulence reversion.

The aim of this study is to construct a genetically stable attenuated ORFV mutant that can be used as a safer and efficient vaccine candidate strain by deleting genes presumably involved in ORFV immunomodulatory properties. We herein described the generation of two single-gene-deletion (*cbp* and *gif*) ORFV recombinants based on a natural strain and then we investigated the effect of these deletions in ORFV replication and virulence *in vitro* and evaluated the safety and effectiveness by immune protection test in goats.

## Materials and methods

### Cells and viruses

PGT cell cultures were prepared from a newborn lamb as previously described with slight modifications (23). Briefly, the outer layers of a fresh testis were removed and then the testis was washed with PBS containing 1% antibiotics (10,000 IU/mL of penicillin and 10,000 µg/mL of streptomycin) (Gibco, USA) and shredded into small pieces with ophthalmic scissors. The chopped tissue was digested with 0.25% trypsin (without EDTA) (Sigma, USA) for 30 min at 37°C and then an appropriate amount of Ham’s DMEM/12 Gluta MAX medium (Thermo Fisher Scientific, USA) containing 10% fetal bovine serum (FBS) (Zhejiang Tianhang Biotechnology Co., Ltd, China) and penicillin-streptomycin solution (final concentration 100 IU/mL of penicillin and 100 µg/mL of streptomycin) was added to neutralize the trypsin. The resulting mixture was centrifuged at 1,000 rpm for 2 min to remove the suspension and the pellet was resuspended in the growth medium supplemented with 10% FBS and penicillin-streptomycin solution and cultured at 37°C under 5% CO_2_.

An ORFV isolated from Gansu Province, China, designated as GS14, was used as a parent virus throughout the experiment and as a genetic background virus to generate target gene-deleted virus. Virus stocks were propagated in PGT cells, and stored in -80°C.

A strain of wild type ORFV obtained from goat infected with ORFV, was used to challenge the immunized animals. The crusted scab of diseased animal was collected and ground with 10 volumes (w/v) of PBS containing 10% antibiotics (10,000 IU/mL of penicillin and 10,000 µg/mL of streptomycin) followed by freeze-thaw cycles. And then the homogenate was centrifuged at 3,500 rpm for 10 min to collect the suspension, which was then filtered through 0.45 μm membrane. PCR method according to the previous study (24, 25) was used to confirm no infection with other common pathogens in sheep and goats, such as bluetongue virus, foot-and-mouth disease virus, *peste des petits* ruminants virus, vesicular stomatitis virus and *Mycoplasma capricolum* subsp. *Capripneumoniae*. And the minimum challenge virus dose was determined by using a series dilution (10^0^, 10^-1^, 10^-2^, 10^-3^) of the wild virus suspension. The main virulence genes of this wild virus strain were identified by PCR and the primers used were shown in **Table 1**. The homology between the wild virus strain and ORFV-GS14 was analyzed by aligning the conservative gene B2L. Plasmids pEGFP-N1 and pcDNA3.1-Cre were kindly provided by China Institute of Veterinary Drug Control.

**Table 1:** Primers used to generate and identify the recombinant viruses.

Primers	Sequence (5’-3’)	Descriptions
cbp-hm1 F	CAGCAAAAAAGGCGAAGTGTT	Used to amplify the left arm of cbp
cbp-hm1 R	TGACCCCGTAATTGATTACTATTAATAACTTCGTATAGCATACATTATACGAAGTTAT CTGAAGCCAGAACCTGGCGT	
cbp-gfp F	ACGCCAGGTTCTGGCTTCAGATAACTTCGTATAATGTATGCTATACGAAGTTAT TAATAGTAATCAATTACGGGGTCA	Used to amplify the EGFP marker gene
cbp-gfp R	TCGCCGTCTCCATATTCCCCATAACTTCGTATAGCATACATTATACGAAGTTATACGCCTTAAGATACATTGATGAGT	
cbp-hm2 F	CACTCATCAATGTATCTTAAGGCGTATAACTTCGTATAATGTATGCTATACGAAGTTATA GGGGAATATGGAGACGGCGA	Used to amplify the right arm of cbp
cbp-hm2 R	TGGCAGGGCGCGACCACGGTCT	
gif-hm1 F^#^	ACCCTATCCTCCTCGGAATGAGC	Used to amplify the left arm of gif
gif-hm1 R^#^	ACGTAGAAGACCAGGAAACAGATAACTTCGTATAGCATACATTATACGAAGTTATCGCGCCGAGTGCACGCTCC	
gif-gfp F^#^	GGAGCGTGCACTCGGCGCGATAACTTCGTATAATGTATGCTATACGAAGTTAT CTGTTTCCTGGTCTTCTACGT	Used to amplify the EGFP marker gene
gif-gfp R^#^	GTAGTCGGTCCCCGGGATTACCATAACTTCGTATAGCATACATTATACGAAGTTATACGCCTTAAGATACATTGATGAGT	
gif-hm2 F^#^	ACTCATCAATGTATCTTAAGGCGTATAACTTCGTATAATGTATGCTATACGAAGTTATAGGTAATCCCGGGGACCGACTAC	Used to amplify the right arm of gif
gif-hm2 R^*^	GATTGGGAACAATTGGGAACG	
gif2-hm1 F^*^	ACCCTATCCTCCTCGGAATGAGC	Used to amplify the left arm of gif
gif2-hm1 R^*^	ACGTAGAAGACCAGGAAACAGCGCGCCGAGTGCACGCTCC	
gif2-gfp F^*^	GGAGCGTGCACTCGGCGCG CTGTTTCCTGGTCTTCTACGT	Used to amplify the EGFP marker gene
gif2-gfp R^*^	GTAGTCGGTCCCCGGGATTACCACGCCTTAAGATACATTGATGAGT	
gif2-hm2 F^*^	ACTCATCAATGTATCTTAAGGCGTGGTAATCCCGGGGACCGACTAC	Used to amplify the right arm of gif
gif2-hm2 R^*^	GATTGGGAACAATTGGGAACG	
F1L-F	ATGGATCCACCCGAAATCACG	Used to identify the *F1L* gene in ORFV
F1L-R	TCACACGATGGCCGTGACC	
gif-F	TCCGGTTAACGCACCCATATT	Used to identify the *gif* gene in ORFV
gif-R	ACCCATTGACGCACATGCTA	
cbp-F	GCGTGTACGGTGGCAGGTCTA	Used to identify the *cbp* gene in ORFV
cbp-R	GGAT ATAGTCATCGCTAT	
vir-F	TCAAGCTTCGAATTACAGG	Used to identify the *vir* gene in ORFV
vir-R	AGGGCGAGGAGCTACGAAC	
vIL-10-F	GTAAACGGCCACAAGTACGGT	Used to identify the *vIL-10* in ORFV
vIL-10-R	ATCAACGTACCAGTAAGGACTT	
w-cbp F	TCTACGGCAACGGGTGAT	Used to identify the deletion of *cbp* gene
w-cbp R	CGGCGACGATTCTTTGTG	
w-gif F	GCTCTAGGAAAGATGGCGTG	Used to identify the deletion of *gif* gene
w-gif R	TACTCCTGGCTGAAGAGCG	

^#^primers were used in the construction of rGS14-ΔGIF; *primers were used in the construction of rGS14-ΔCBP-ΔGIF.

### Construction of transfer vectors

For the targeted deletion of the CBP or GIF genomic locus by homologous recombination, two recombinant transfer vectors, comprising a selection marker gene, the upstream and downstream homologous arms flanking the target viral genomic region, were constructed by one-step fusion PCR method. Briefly, left (hm1) and right (hm2) arms flanking each target gene and corresponding selection GFP cassette were amplified by PCR, respectively. The GFP cassettes were amplified based on fragment of pEGFP-N1 and flanked on both sides with LoxP sites. The downstream of hm1 and the upstream of GFP cassette contain a section of complimentary sequence, the same with the downstream of GFP cassette and the upstream of hm2. Then the purified hm1, GFP cassette and hm2 were assembled to generate the recombinant transfer vectors by fusion PCR using primers of hm1 F and hm2 R, designated as pCBPhm1-GFP-CBPhm2 and pGIFhm1-GFP-GIFhm2. Primers used to construct the recombinant vectors were synthesized by Sangon Biotech (Shanghai) Co., Ltd, China (**Table 1**).

### Generation of rGS14-ΔCBP and rGS14-ΔGIF

**Figure 1:**
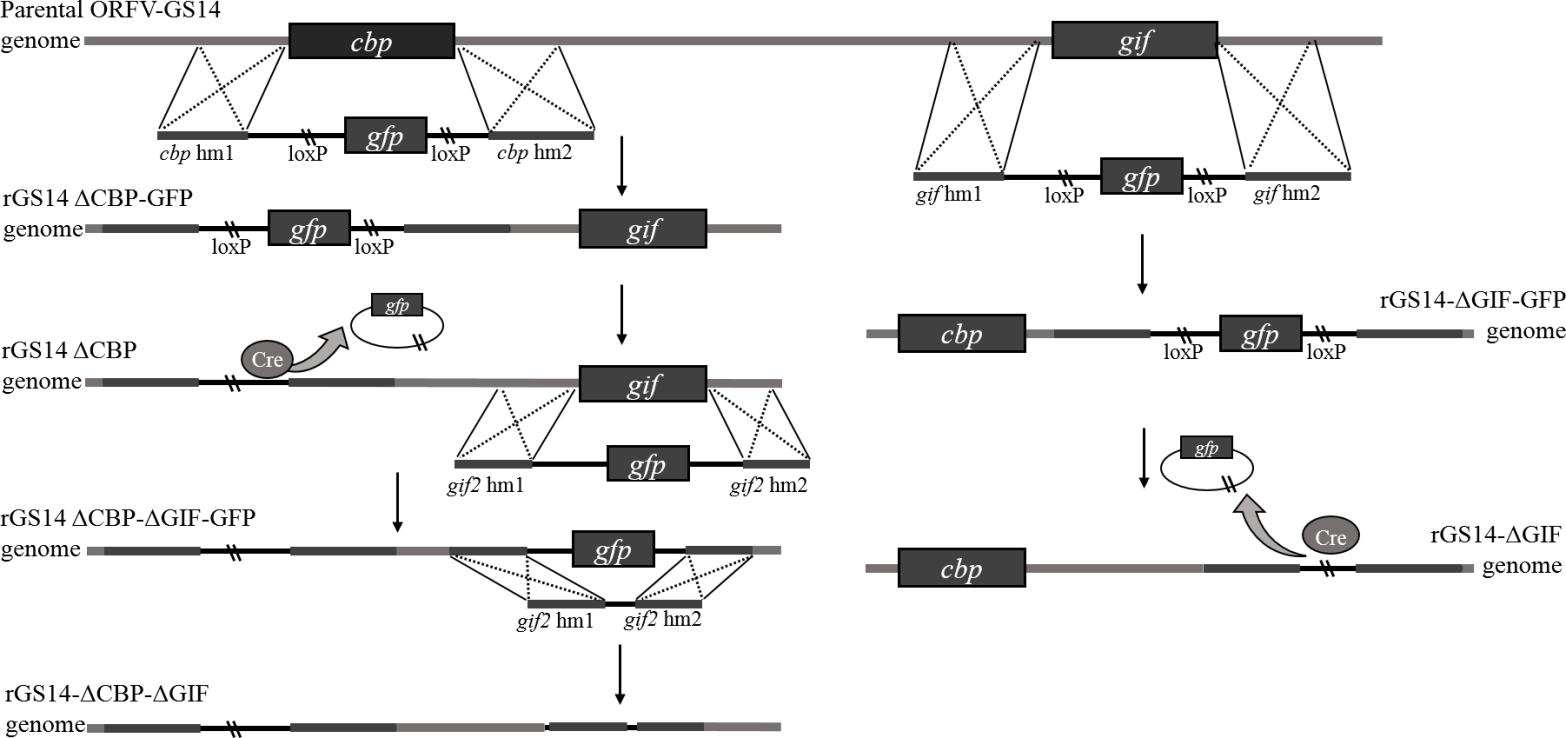
Diagram of homologous recombination between plasmid DNA and genome of parental ORFV.

Recombinant viruses rGS14-ΔCBP-GFP and rGS14-ΔGIF-GFP were generated by homologous recombination between the parental ORFV-GS14 genome (**Figure 1**) and the corresponding recombination transfer vector following infection and transfection of PGT cell cultures as previous described with appropriate modifications (26). Briefly, monolayers of PGT cells were seeded in 6-well plates and were infected with 0.5 mL 10^7.7^ TCID_50_/mL of ORFV-GS14 and subsequently transfected with 4 μg of the transfer vector pCBPhm1-GFP-CBPhm2 and pGIFhm1-GFP-GIFhm2 separately at 24 h post-infection(pi), using 5 μL of Lipofectamine 3000 reagent (Thermo Fisher Scientific, USA) according to the manufacture. The plaques with a GFP signal were selected under fluorescence microscopy and further purified by limiting dilution assay. Genomic DNA was extracted using by AxyPrep body fluid viral DNA/RNA miniprep kit (Axygen, USA). PCR was performed to screen for the wild type virus contamination using internal primers including w-cbp F, w-cbp F, w-gif F and w-gif R (**Table 1**).

The reporter *gfp*-gene in rGS14-ΔCBP-GFP and rGS14-ΔGIF-GFP was deleted by Cre-loxP recombinase system according to the manufacturer’s instructions. Briefly, PGT cells were infected with rGS14-ΔCBP-GFP and rGS14-ΔGIF-GFP, respectively. 24 h later, the cells were transfected with plasmid pcDNA3.1-Cre as mentioned above and were incubated until showing evident cytopathic effect (CPE). Plaques without GFP signal were picked to make virus stock and then passaged several times in PGT cells and purified further by limiting dilution. The excision of GFP from the viruses by Cre recombinase was confirmed by PCR of the GFP cassette using primers cbp-hm1 F, cpb-hm2 R and gif-hm1 F, gif-hm2 R, respectively.

### Generation of rGS14-ΔCBP-ΔGIF

In order to get a double gene-deleted recombinant virus rGS14-ΔCBP-ΔGIF-GFP, the *GIF* gene was deleted from the *cbp*-deleted rGS14-ΔCBP as described above. For the deletion of *gfp* gene of the rGS14-ΔCBP-ΔGIF-GFP, a recombinant transfer vector without LoxP sites or reporter gene cassette containing left and right recombinant arms flanking the *gif* gene was constructed as described above. And the final double gene-deleted recombinant virus was generated by homologous recombination as described above and designed as rGS14-ΔCBP-ΔGIF. After successive purification, the genomic DNA of rGS14-ΔCBP-ΔGIF was extracted by AxyPrep body fluid viral DNA/RNA miniprep kit (Axygen, USA) and the deletion of both CBP and GIF genes were confirmed by PCR using the primers (**Table 1**). The genomic DNA of GS14 was used as a control.

### Virus titration, and growth curve

#### TCID_50_ titration

The virus titers of rGS14-ΔCBP-ΔGIF in PGT cells were determined and expressed as tissue culture infection dose 50 (TCID_50_) per mL, which referred to previous study with slightly modification (5). Briefly, PGT cells were infected with serial 10-fold dilutions of mutant viruses of 10^-1^ to 10^-12^. After 2 hours of adsorption at 37°C under 5% CO_2_ the inoculum was removed, and the cells were rinsed two times with PBS. The monolayers were then maintained with growth medium at 37°C under 5% CO_2._ The wells with CPE were counted at 7^th^ day post-infection and TCID_50_ was calculated as Reed-Muench methods.

To investigate the difference of plaque morphology between rGS14-ΔCBP-ΔGIF and parental ORFV-GS14, same dose of ORFV-GS14 was used to infect PGT cells and CPE characteristics were recorded as mentioned above.

### Single-step growth curve and genetic stability analysis

Replication properties of rGS14-ΔCBP-ΔGIF and parental ORFV-GS14 were assessed in PGT cell cultures. Preformed monolayers were prepared in 6-well plates and infected with the recombinant virus and parental virus at a MOI of 1, respectively. Then the cells were incubated at 37°C under 5% CO_2_ and harvested at various time points (6, 12, 24, 36, 48, 60, 72, 84, 96 and 108 h post infection) followed by repeated freezing and thawing at -80°C. The thawed lysates were used to determine titers by TCID_50_ in PGT cells and virus load was qualified on each time point by using qPCR method.

To investigate the genetic stability of rGS14-ΔCBP-ΔGIF, serial passages of the virus were conducted in PGT cells. Low MOI (~1) infections were performed with the recombinant virus and cells were harvested at 48 h pi. Passages 1, 5, 10 and 15 viruses were subjected to PCR assays to evaluated the stability of the *cbp* and *gif* gene deletion.

### Animals and virus inoculation

In order to assess the virulence of the gene deleted recombinant viruses in goat, twenty 1-month-old lambs with ORFV antibody free were divided into four groups of five animals each (n=5), and inoculated with each of three ORFV-recombinants (rGS14-ΔCBP, rGS14-ΔGIF and rGS14-ΔCBP-ΔGIF) or PBS. The lambs in the trial group were inoculated with 200 μL of a virus suspension containing 10^6^TCID_50_/mL in the labial commissures. Virus inoculation process referred to the method described previously (16). The control group was treated with 200 μL PBS after scarification.

In challenge experiments, animals were inoculated with 200 μL of the three recombinant virus suspension (10^5^TCID_50_/mL) respectively and then were challenged with 100 TCID_50_ of orf virus on the 21^st^ day post-inoculation.

### Clinical and virological monitoring

All the lambs were monitored daily for 21 days post-inoculation of the virus. The body temperature was one of the main signs and must be monitored and the clinical scoring was performed according to Mittelholzer’s and Martins’ method (27, 28) with modifications. Briefly, clinical scoring was conducted by two examiners who were blinded to the experimental groups and six parameters were employed for evaluation, including anorexia, depression, fever, hyperemia, vesicles and/or pustules, and scabs. Each parameter was scored from 0 to 3, and the higher number means worse situation. The clinical score for each animal was calculated and then the mean clinical score and the standard error of the mean for each group were calculated [mean ± standard error of mean (SEM)].

In challenge experiments, the animals were continuously monitored for 14 days post-challenging and clinical photographs and data were collected.

## Results

### Generation of recombinant viruses

**Figure 2:**
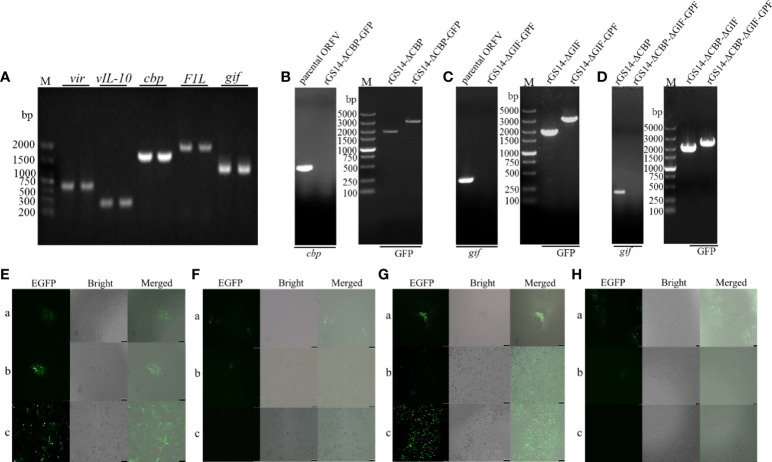
Construction of Diagram of gene-deletion recombinant ORFV. **(A)** Agarose gel demonstrating PCR amplification of the main conservative gene and virulent genes from the genome of the parental ORFV-GS14. **(B)** Representative agarose gel demonstrating the deletion of *cbp* gene from parental ORFV-GS14 and the generation of rGS14-ΔCBP. **(C)** The same experiments for the recombinant viruses rGS14-ΔGIF and **(D)** rGS14-ΔCBP-ΔGIF. **(E)** Plaque purification of rGS14-ΔCBP-GFP. a: the first round of plaque purification, b: the third round of plaque purification, c: the sixth round of plaque purification. **(F)** Plaque purification of rGS14-ΔCBP. a: the second round of of plaque purification, b: the third round of plaque purification, c: the fourth round of plaque purification. **(G, H)** the same experiments for the recombinant viruses rGS14-ΔCBP-ΔGIF-GFP and rGS14-ΔCBP-ΔGIF.

The main conservative gene and virulence genes of the parental ORFV-GS14 were identified by PCR, and agarose gel electrophoresis confirmed the existence of *vir*, *vIL-10*, *cbp*, *gif* and *F1L* gene in the genome of ORFV-GS14 (**Figure 2A**). Using homologous recombination, the *cbp* and *gif* gene sequences were deleted from the parental ORFV-GS14 genome and replaced by the GFP reporter gene sequences, respectively (**Figures 2B, C**). After six rounds of plaque purification, the amount of single gene deletion recombinant viruses with GFP (rGS14-ΔCBP-GFP and rGS14-ΔGIF-GFP) were accumulated (**Figure 2E**). Delated *cpb*, or *gif* gene were not detected in the purified recombinant viruses rGS14-ΔCBP-GFP and rGS14-ΔGIF-GFP, respectively, but could be detected in the parental ORFV-GS14 (**Figures 2B, C**). The *gfp* gene in the two viruses was then knocked out by LoxP system (**Figures 2B, C, F**), generating two single gene-deleted recombinant viruses rGS14-ΔCBP and rGS14-ΔGIF, respectively. rGS14-ΔCBP was used as a second parental virus to generate recombinant virus rGS14-ΔCBP-ΔGIF with the double gene deletion by homologous recombination. The *gif* gene in the genome of rGS14-ΔCBP was replaced by *gfp* to generate rGS14-ΔCBP-ΔGIF-GFP after six rounds of screening (**Figures 2D, G**). Afterwards the GFP reporter gene was deleted by homologous recombinant between a recombinant transfer vector containing the left arm and right arm of *gif* gene and the genome of rGS14-ΔCBP-ΔGIF-GFP. The double gene recombinant virus rGS14-ΔCBP-ΔGIF were obtained after successive purification and the knocking out of *gfp* gene was confirmed by fluorescence microscope and PCR (**Figures 2D, H**). Genome sequencing of rGS14-ΔCBP, rGS14-ΔGIF and rGS14-ΔCBP-ΔGIF confirmed the deletion of *cbp* and/or *gif* gene and the homology with parental ORFV-GS14. Besides the deletion of *cbp* and/or *gif* gene, no significant additional differences were observed across the entire genome of these viruses (data not shown).

### *In vitro* characterization of the recombinant virus

**Figure 3:**
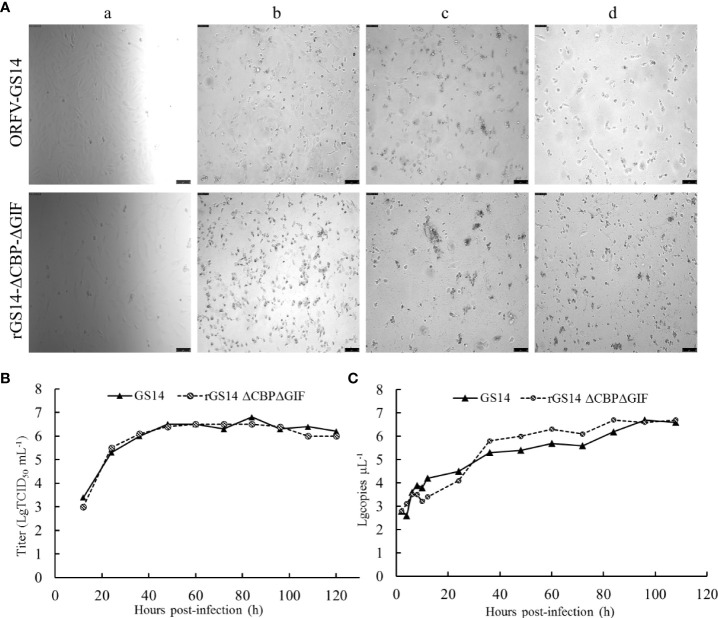
Plaque morphology and *in vitro* replication of rGS14-ΔCBP-ΔGIF compared to parental ORFV-GS14. **(A)** The plaque morphology differences between rGS14-ΔCBP-ΔGIF and parental ORFV-GS14 at different time points post-infection. a: 10 h post-infection; b: 48 h post-infection; c: 72 h post-infection; d: 96 h post-infection. **(B)** Single-step growth curve (MOI = 1) of rGS14-ΔCBP-ΔGIF in PGT cells. **(C)** Virus quantity of rGS14-ΔCBP-ΔGIF in PGT cells at different time points post-infection. The copy numbers of the virus at different time points post-infection were determined by real-time quantitative PCR. Results were calculated based on two independent experiments.

Replication properties of rGS14-ΔCBP-ΔGIF were compared to the parental ORFV-GS14 in PGT cells. The TCID_50_ of rGS14-ΔCBP-ΔGIF was 10^-6.3^/0.1 mL which was approximate to that of the parental ORFV-GS14 (10^-6.7^/0.1 mL). And the plaque size and morphology produced by the double gene deleted recombinant virus and parental virus at different time points post infection showed no significant differences (**Figure 3A**). The *in vitro* growth kinetics of rGS14-ΔCBP-ΔGIF was evaluated in PGT cell cultures at MOI of 1 and results demonstrated that rGS14-ΔCBP-ΔGIF exhibited similar growth kinetics to the parental virus (**Figure 3B**). The genome DNA of viruses recovered at different hours post-infection were extracted and analyzed by real-time quantitative PCR, respectively. Virus quantity curve of rGS14-ΔCBP-ΔGIF and parental ORFV-GS14 also presented similar tendency (**Figure 3C**), which was consistent with the single-step growth curve.

### The genetic stability of rGS14-ΔCBP-ΔGIF

The stability of *cbp* and *gif* gene deletion was assessed by PCR following serial passages of the recombinant virus in PGT cells *in vitro*. The deficiency of *cbp* and *gif* gene was consistently detected in rGS14-ΔCBP-ΔGIF infected cells after 1, 5, 10 and 15 passages of the recombinant virus in cell cultures. PCR amplification of *cbp* and *gif* from their own locus of the genome of passages 1, 5, 10 and 15 rGS14-ΔCBP-ΔGIF confirmed the stability of *cbp* and *gif* gene deletion (data not shown).

### Biosafety assessment of rGS14-ΔCBP-ΔGIF

**Figure 4:**
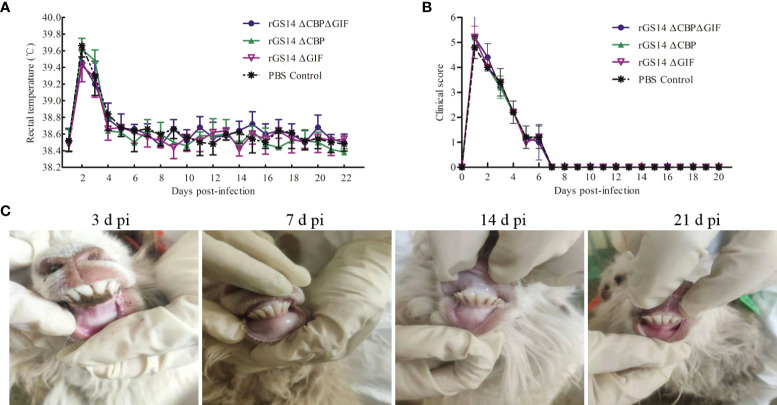
Biosafety assessment of rGS14-ΔCBP, rGS14-ΔGIF and rGS14-ΔCBP-ΔGIF. **(A)** The rectal temperature of lambs inoculated with rGS14-ΔCBP, rGS14-ΔGIF, rGS14-ΔCBP-ΔGIF or PBS from the 1st to 21^st^ day post-inoculation. The bars represent the standard error of the mean. **(B)** Mean clinical score in lambs inoculated with rGS14-ΔCBP, rGS14-ΔGIF, rGS14-ΔCBP-ΔGIF or PBS from the 1st to 21^st^ day post-inoculation. The bars represent the standard error of the mean. **(C)** The cut healed on 5 to 7 days post inoculation and no pathological changes was observed in all the animals during 21 days post-inoculation. d pi = days post-inoculation.

To assess whether the deletion of the *cbp* and *gif* genes makes rGS14-ΔCBP-ΔGIF a safe candidate vaccine strain, lambs were inoculated with 200 μL of 10^6^TCID_50_ rGS14-ΔCBP-ΔGIF following scarification of the oral commissures and monitored daily for 21 days. The virulence of rGS14-ΔCBP and rGS14-ΔGIF was also evaluated. All the inoculated lambs did not present any clinical sign associated with ORFV, and the cut healed on 5 to 7 days post-inoculation. No maculo-papular, ulcer and proliferative scabby lesions was observed in the skin of the lips, around the mouth or nostrils and oral mucosa during 21 days post-inoculation (**Figure 4C**), indicating that the gene deleted recombinant viruses become attenuated or less able to cause disease at the inoculated dose.

However, all lambs inoculated with rGS14-ΔCBP, rGS14-ΔGIF, rGS14-ΔCBP-ΔGIF or PBS had slightly increased body temperature (>39.4) by day 2 post-inoculation and then the temperature fell to and maintained the normal level from day 5 to 21 post-inoculation (**Figure 4A**). Additionally, the clinical score curve of all animals displayed a similar tendency relative to the changes of body temperature (**Figure 4B**). All animals inoculated with the recombinant viruses or PBS presented clinical signs including anorexia, depression and fever resulting in the increase of clinical score from day 1 to 6 post-inoculation and the clinical symptoms subsided on day 8 post-inoculation. However, the clinical signs were not associated with ORFV since vesicles, pustules and scabs were not observed at the oral commissure.

### Protective efficacy of rGS14-ΔCBP-ΔGIF against wild virulent ORFV strain challenge in lambs

**Figure 5:**
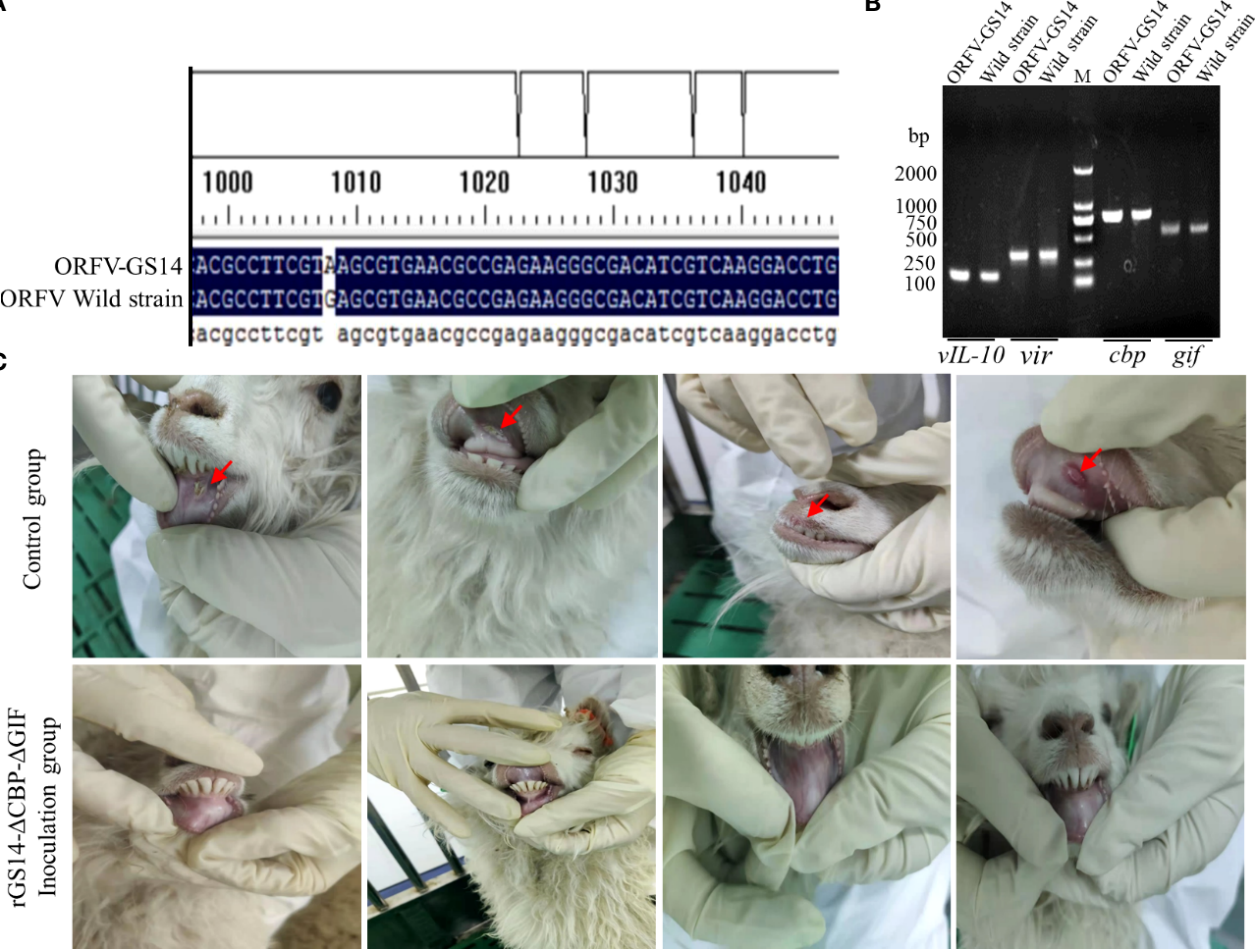
Identification of a wild ORFV used to challenge and the protective efficacy of rGS14-ΔCBP-ΔGIF. **(A)** The existence of main virulence genes was confirmed in the genome of the wild ORFV. **(B)** Sequencing analysis of the wild ORFV compared with ORFV-GS14. **(C)** Lambs inoculated with rGS14-ΔCBP-ΔGIF were all remained healthy compared with the control group.

A wild type ORFV obtained from proliferative scabby lesions tissues of infected goat was used to assess the ability of rGS14-ΔCBP-ΔGIF inoculation to induce protection against challenge with virulent ORFV. The existence of main virulence genes including *vIL-10*, *vir*, *cbp* and *gif* in the genome of the wild ORFV were confirmed by PCR method (**Figure 5A**). The results of sequencing analysis based on the B2L gene demonstrated that the nucleotide of wild ORFV shared homology of 97.09% with that of ORFV-GS14 strain (**Figure 5B**). And the minimum pathogenic dose of the wild ORFV suspension was 10^-2^ dilution.

Lambs in the trial group were inoculated with 200 μL of 10^5^TCID_50_ rGS14-ΔCBP, rGS14-ΔGIF and rGS14-ΔCBP-ΔGIF and the control group received equal volume of PBS. Twenty-one days later, all animals were challenged with 10^-2^ dilution of the wild virulent ORFV after skin/mucous membrane scarification of the oral commissures. All animals in the control group showing ORFV associated clinical symptoms including oral ulcer, pustules, redness and swelling and slight scabs by 3 to 4 days post-challenging (**Figure 5C**). While, 3 lambs inoculated with rGS14-ΔCBP and 2 lambs inoculated with rGS14-ΔGIF presented ORFV related signs after challenging, which showed an immune protection rate of 40% and 50%, respectively (**Table 2**). The group of animals inoculated with rGS14-ΔCBP-ΔGIF all remained clinically healthy, not exhibiting any significant signs of disease during the 14-day observational period (**Figure 5**) except anorexia and a slight fever by day 5 post-infection. The double gene deleted rGS14-ΔCBP-ΔGIF provided a complete protection to animals that were challenged by virulent ORFV at a minimum pathogenic dose.

**Table 2:** Protective efficacy of gene deleted recombinant viruses.

Group	*n*	No. of animals presenting disease related signs after challenging	Protection rate
rGS14-ΔCBP	5	3	40%
rGS14-ΔGIF	4	2	50%
rGS14-ΔCBP-ΔGIF	4	0	100%
PBS	4	4	–

Protection rate=number of animals without ORFV related symptoms/total number in each group.

## Discussion

ORFV has a worldwide distribution resulting in significant economic losses (29). Although live attenuated virus and attenuated tissue culture vaccines are useful in reducing the disease severity and taken into practice where contagious ecthyma infections are endemic, these vaccines can disseminate the vaccine strain capable of causing the disease and unable to confer solid immunity to re-infection (30). The attenuated virus strains by genetic manipulation is considered a promising alternative to develop an effective contagious ecthyma vaccine and is safer than the naturally attenuated isolates. And attenuated strains of other intracellular pathogens obtained by gene deletion have been proved to induce protection against the virulent viruses (31, 32). Various virulence-associated genes of ORFV have been identified, and the function of these genes has been clarified (10, 16 and 26). Based on the pre-determined genetic factors, several single gene-deleted recombinant ORFV strains were generated. However, the virulence, immunogenicity, safety, and protective efficacy of these viruses remains concerns. According to the previous study, a single-gene deletion only resulted in slight reduction in virulence *in vivo* (16), and the incompletely attenuated virus is not a good choice for vaccine use.

In this study, we constructed a double gene-deleted recombinant virus rGS14-ΔCBP-ΔGIF based on the ORFV strain GS14, which was originally isolated from a diseased sheep. As described above, CBP and GIF are two important IMPs that contribute to ORFV virulence playing a vital role in the infection progress of ORFV. A recent study demonstrated that *cbp* and *gif* gene are non-essential for ORFV replication *in vitro* and the individual deletion of *cbp* and *gif* genes from the genome of ORFV did not affect the ability of the recombinants to replicate in primary ovine fetal turbinate cells (16). In addition, this study also investigated the effects of *cbp* or *gif* gene deletion in ORFV biology and pathogenesis *in vivo*. It was found that the deletion of *cbp* or *gif* gene from the genome of ORFV IA82 strain only resulted in slight reduction in virulence in lambs, which was inconsistent with the Fleming’s results (18). Nonetheless, no reports about the effect of double gene deletion of *cbp* and *gif* gene on the virulence of ORFV were found to date. Herein, we targeted *cbp* and *gif* gene and double gene knockout were conducted from the genome of ORFV-GS14. Characterization *in vitro* of the recombinant rGS14-ΔCBP-ΔGIF revealed no difference in TCID_50_, plaque morphology or the replication kinetics comparing parental strain ORFV-GS14. These results implied that double deletion of *cbp* and *gif* gene had little impact on the growth kinetics of ORFV as well, which corresponded to the previous conclusions that *cbp* and *gif* gene was not responsible for the replication of ORFV. Moreover, the double gene-deleted rGS14-ΔCBP-ΔGIF showed good genetic stability by serial passages *in vitro*, which is conducive to the large-scale preparation of the recombinant virus strain.

Homologous recombination has become a mature method to generate gene deletion virus strains. And the transfer vector containing homologous arms flanking the target gene is the most widely used tool during the process. It was reported that the efficiency of homologous recombination was closely related to the length of homology arms and at least 30 bp of homology was required for a relatively high efficiency (33). On the other hand, linear DNA is more easily integrated into the genome of host cells than circular DNA after transfection. Consequently, the homologous recombinant arms constructed in this study was linear DNA with a length of about 1 kb. The host cell for constructing rGS14-ΔCBP-ΔGIF is another important factor that may affect transfection efficiency. According to Gülyaz’s results (22), ORFV vaccine strain was adapted to Madin-Darby Bovine Kidney (MDBK) cell cultures well so that MDBK cell was initially selected as the host cell in our study. However, low transfection efficiency of the homologous recombinant vector in MDBK cells led to a low titer of recombinant virus, unable to screen and purify the positive clones. Luckily, we found that the PGT cell was more suitable for transfection of recombinant vector than MDBK cells after many attempts. Hence, the PGT cell cultures were used in the progress of generating the rGS14-ΔCBP-ΔGIF.

Safety and reliability are the basic requirements of veterinary biological products. In the present study, rGS14-ΔCBP-ΔGIF was constructed by knocking out two important virulence genes based on the natural attenuated strain, which can minimize the risk of virulence return of the recombinant virus. The safety of the recombinant viruses was tested in 1-month-old lambs and results demonstrated that no ORFV associated symptoms such as ulcer and proliferative scabby lesions were induced by either rGS14-ΔCBP, rGS14-ΔGIF or rGS14-ΔCBP-ΔGIF at 21 days post-infection. In the safety experiment, slight fever and anorexia were observed at 3 days post-infection leading to the increase of the clinical score (**Figures 4A, B**), for which the major reason was speculated to be the cut in the labial commissures and stress response. These results indicated that the virulence of three gene-deleted recombinant virus strains (rGS14-ΔCBP, rGS14-ΔGIF and rGS14-ΔCBP-ΔGIF) based on the natural attenuated ORFV-GS14 was markedly attenuated.

The immune-protection effect of rGS14-ΔCBP, rGS14-ΔGIF and rGS14-ΔCBP-ΔGIF was evaluated by challenging the inoculated lambs with a wild virulent ORFV strain. Results showed that the double gene-deleted strain rGS14-ΔCBP-ΔGIF effectively protected 100% of the lambs (4/4) when challenged at 21-day post-inoculation, while the single gene-deleted strains, rGS14-ΔCBP and rGS14-ΔGIF, protected only 40% (2/5) and 50% (2/4) of the animals respectively, with lambs in the control group all infected. The protective efficacy of the double gene-deleted strain was significantly better than that of the single-gene deletion strains. We suspected that the difference of the protection rate between rGS14-ΔCBP-ΔGIF and two single gene-deleted strains was caused by the effect of *cbp* or *gif* gene. As reported, CBP inhibits the recruitment of dendritic cells (DC) or their precursors from the blood into the skin as well disrupting chemokine gradients that regulate the migration of activated DC to peripheral lymph nodes (34). As a central mediator of tissue inflammation, GIF is involved in the stimulation of monocytes and granulocytes for the initial immune response and capable of stimulating DC for priming the adaptive immune response (35). The *gif* or *cbp* gene remained in the genome of the single gene-deleted recombinant viruses contributed the immune escape of them *in vivo*, while the double deletion of *cbp* and *gif* genes resulted in a negative effect on immune escape so that rGS14-ΔCBP-ΔGIF could be more easily recognized and remembered by the immune system.

In summary, in this study, we successfully generated a double gene-deleted ORFV, rGS14-ΔCBP-ΔGIF, by series homologous recombination method and evaluated its safety and protection potency in lambs. The results indicated that rGS14-ΔCBP-ΔGIF provided completely protection against wild virulent ORFV challenge and would be a promising candidate vaccine strains with safety and availability.

## Data availability statement

The original contributions presented in the study are included in the article/**Supplementary Material**. Further inquiries can be directed to the corresponding author.

## Ethics statement

This study was reviewed and approved by the Animal Care and Use Committee of China Institute of Veterinary Drug Control and China Institute of Veterinary Drug Control.

## Author contributions

ZZ: design the experiment and provided the techniques for the gene deletion. GQ, CW and ZYZ: literature review and interpretation, manuscript writing. JD, YC and ZS: Generate recombinant viruses and characterize the recombinant virus *in vitro*. Cell culture and virus preparation. CY: animal experiments. XC: design the experiment and did the final approval of manuscript. All authors contributed to the article and approved the submitted version.

## Funding

This work was supported by grant from the National Key Research and Development Program of China (No. 2016YFD0500906)

## Conflict of interest

The authors declare that the research was conducted in the absence of any commercial or financial relationships that could be construed as a potential conflict of interest.

## Publisher’s note

All claims expressed in this article are solely those of the authors and do not necessarily represent those of their affiliated organizations, or those of the publisher, the editors and the reviewers. Any product that may be evaluated in this article, or claim that may be made by its manufacturer, is not guaranteed or endorsed by the publisher.
