# Supplementary material for: Construction and characterization of a contagious ecthyma virus double-gene deletion strain and evaluation of its potential as a live-attenuated vaccine in goat
Source: Front Immunol. 2022 Sep 2;13:961287. doi: 10.3389/fimmu.2022.961287 (PMC9478544; doi:10.3389/fimmu.2022.961287)
Supplement: Supplementary file 1 [file DataSheet_1.docx]

**1. The sequence of gif, cbp, vegf and vir gene and their BLAST results were provided as following:**

**gif sequence:**

TAGGCCGCGCTGTGCGGAGCGTGCACTCGGCGCGATGGATCGGCGAGCGCGACTTCTGCATGGCCCACGCGCAGGACGTCTTCGCTCGGCTGCAGGTGTGGATGCGCATCGACCGAAACGTGACCGCCGCGGACAACAGCTCGGCCTGCGCGCTGGCGATAGAGACGCCGCCGAGCAACTTCGACGCGGACGTCTACGTCGCCGCGGCCGGCATAAACGCCAGCGTGTCCGCGATCAACTGCGGCTTCTTCAACATGCGCCAGGTAGAGACAACGTACAACACGGCACGCCGGCAGATGTACGTCTACATGGACACCTGGGACCCCTGGGTGCTCAACGCCCCCCAGCCGCTCTTCAGCCAGGAGCACGAAAACGAAACGCTGCCGTACCTGCTGGAGGTTCTAGAGCTAGCGAGGCTGTACATTCGCGTGGGCTGCACGGTGCCCGGAGAGCAGCCCTTTGAGGTGATCCCGGGGATCGACTACCCCCACACCGGCATGGAGTTTCTCCAGCACGTTCTACGGCCAAACCGCCGGTTCGCTCCAGCGAAGCTGCACATAGACCTCGAGGTGGACTACCGGTGCGTGAGCGCCGTCCACGTGAAGGCGTTCCTGCAGGACGCCTGTAGCGCCCGCAAGGCGCGGACGCCACTCTACTTCGCGGGGCATGGCTCCAACCATCCAGATCGCCGGCCAAAAAACCCAGTACCGCGCCCTCAGCATGTGTCGTCACCGATGTCCAGGAAGTGCTGCATGCAGAGTGACGACTGACCTAAA

**BLAST results:**


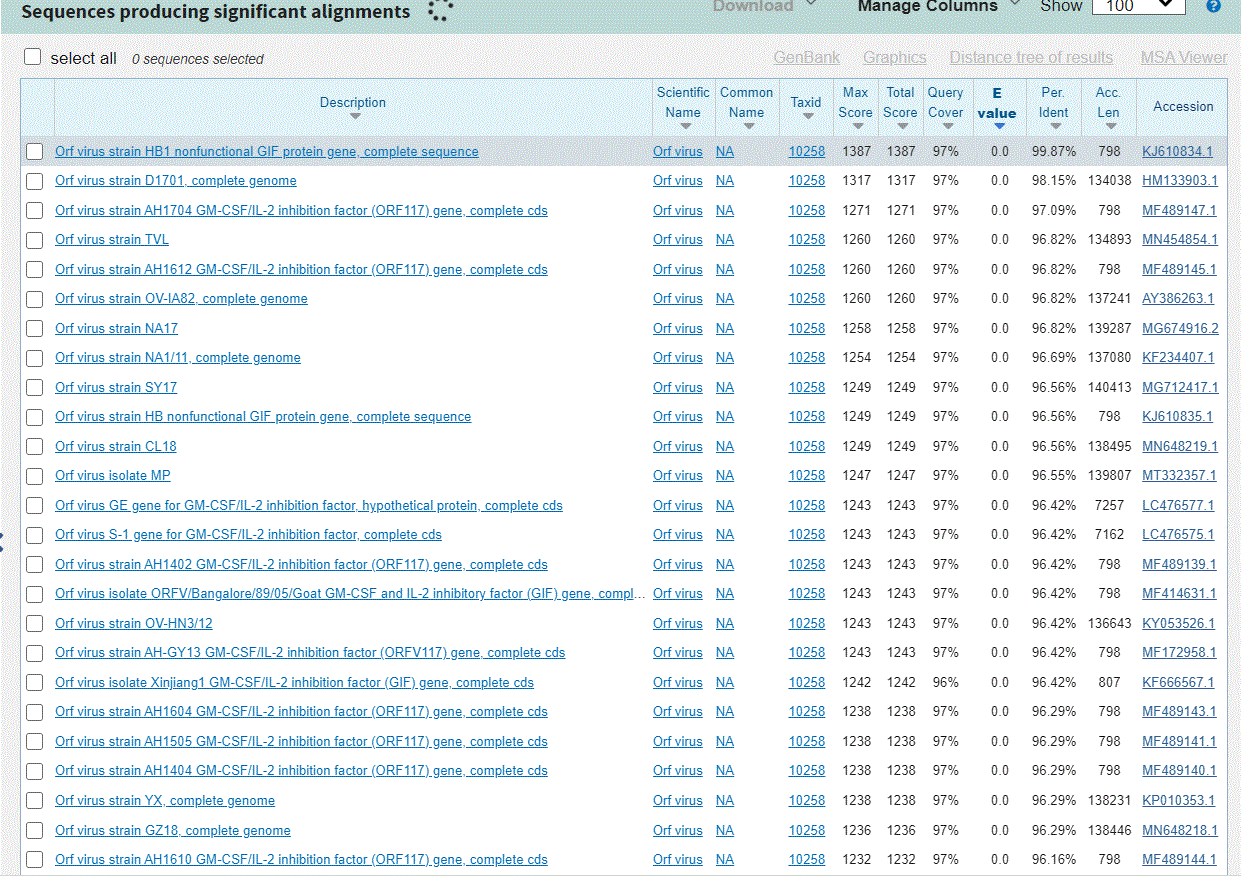


**cbp sequence**

CAGCAAAAAAGGCGAAGTGTTTAAGTCTTTAGGCGAAACAGCTAAAATTTGGAACGGTGCTGGTGCTGGCCAACAGGAAAAAGAATTCTTTTGTGTTACAAAACTTTAATCTCTAATAAATGGGTTGTTGCAGGACACCGAATAGACAGTGCTTGCTAACGCTAAAGCGGGCCTCGTGCCCCGTAGCGTCTCTCGTGTCTGTGCTATCCGTGTTTACTAGCATCTGCGCCATTATCAAGTATACTGACCTTTTTCTAAAAGAAGCGTGTGAAAACGATTGGGTTCCCATTAAAGACCTTTGCGTGTATAACACTCAAGTTATGACTAATCTTACGTTGGCGAAAGATATATGTGCTTCTATGGACAGCGACCTTCCTGCTACACCAGACACTATGTTTCTAAGAGGGATAATGTTTATCGTAGAAGCTACCAGCTTTTGGATGACGCACCACGATGCATATAGAAACGTATATTTGCCAAAAAGAGGAAATAGTCATTTAGGGGCTTATGTGGAGTACGACAAAGACACACACGTGTGTCTTATAAACCTGCAAGGGCTTATGCATCACGATTGTAATCAAAACACCACTGTTGTCTGCGTAAAAAAGATGTACAATAACTGAAAATATACTGTTTGAACGCAAAGACGCCATGTCGCGACTTCAAATACTGACCTCATTTGGACAAATCTACGCACCTGACGAAGCTCGGCTGCGAGAGATCGCGCGTGATTTGGGAATATGCACCATAAAACGCGCATTCGGCGACATGCTGTACGGCTTTATAGACTTCAACCCGGTGCCCCTGACCCAAGTAAACATGCTCATGCCCAACTGCTACTTCGCGGTCAACGGCAACCTGCTTCCGTGCACGGAGGACTTCCGGCTCAGACTCCCGGCAACGGAGATCTCTGCGGCCTACCTGACGAAAACGGGACGGACGATCCTGTGCGGTAGAGACTTCAACATAGTAGCGCCGTCGGGGTTCAAGCGCAGAAGAAGAAAAATTCTGCTCGACGCATCAGGGCGAAGTGCACGCCAGGTTCTGGCTTCAGATCTCTGCGGCCTACCTGCGAAAACGGGACGGACGATCCTGTGCGGTAGAGACTTCAACATAGTAGCGCCGTCGGGGTTCAAGCGCAGAAGAAGAAAAATTCTGCTCGACGCATCAGGGCGAAGTGCACGCCAGGTTCTGGCTTCAGATGCGCGTGGGTGTACGACACAGTCCGCTCTACACTCCCAGCAACATGTGCATGATGGACATAGAAGACTCTACGGACACAGAAGACTCCACGATGGAAAAAGAATACACGTCTACGGCAACGGGTGATGCGGACGGATTGAACGTGTCCGTAGCACTAATTGGAGAAGGCGTGAGCATACCGCTAAGTTACATAGGCCTTAGATTTAACCCGTCGCTTACAGATGGCTACCTGTACGTCAACGTCTCGTCACGGGCTCCTTGGGATCAACAGACTCTGGACCTATCCGCGAACGACGGCTGGGGTATCAAACAGGTTCTAGAAAAAGAGATACTGGCCATCCAGATAGGGTGCGACAACCAAAAATTTCCCGAAGAACCCACAACTACCCAACCCCCCTCACCTGTCACAACAACGCTTTCCTCAACAACTCTAGATCCGAATGACGAAAACACAGACACTACGCCGACAACCACCGGCGACAGTGTAGACGGAAAGCGCAATCCAGATGACTTTGACTTCTCGCTGATCGTGGACCCCCGATGCGTGACCTCTGTAAACCTGCACTTTGAGATTAAGGACGCGTGCATGGACCACAAAGAATCGTCGCCGTTGTCGCTGAAGGGGGAATATGGAGACGGCGAACTAGTAAGAAAAGAAATCAAAAACGTGGGAAAGGATCACAATATGTGCAGTCTTAACCTCAGCCCTGGCCATTGAGCTGTTTTTATTCGGCAATATAATAGGTGATTATTGAACATTAAACAAAACTTATCCCACAACGCCGCAACAATGGAAGTGCTGGTGATCGTCTCTATTATTGTCGCCGTAATATGCTTAACTGGAG

**BLAST results:**


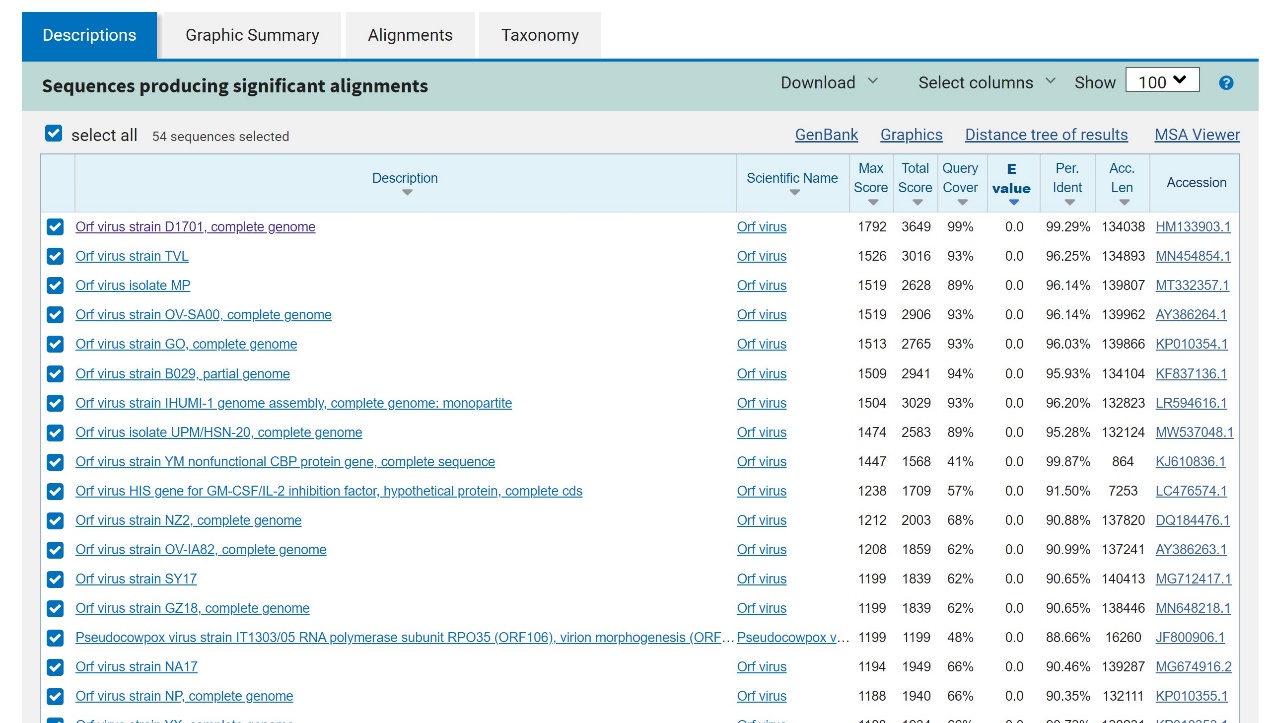


**vegf sequence**

CCGTCGGCATACTAGTAGCCGTGTGCTTGCACCAGTATCTGCTGAACGCGGACAGCAACACGAAAGGATGGTCCGAAGTGCTGAAAGGCAGCGAGTGCAAGCCTAGGCCGATTGTTGTTCCTGTAAGCGAGACGCACCCAGAGCTGACTTCTCAGCGGTTCAACCCGCCGTGTGTCACGTTGATGCGATGCGGCGGGTGCTGCAACGACGAGAGCTTGGAATGCGTCCCCACGGAAGAAGTAAACGTGACGATGGAACTCCTGGGGGCGTCGGGCTCCGGTAGTAACGGGATGCAACGTCTGAGCTTCGTAGAGCATAAGAAATGCGATTGTAGACCACGATTCACAACCACGCCACCGACGACCACAAGGCCGCCCAGAC

**BLAST results:**


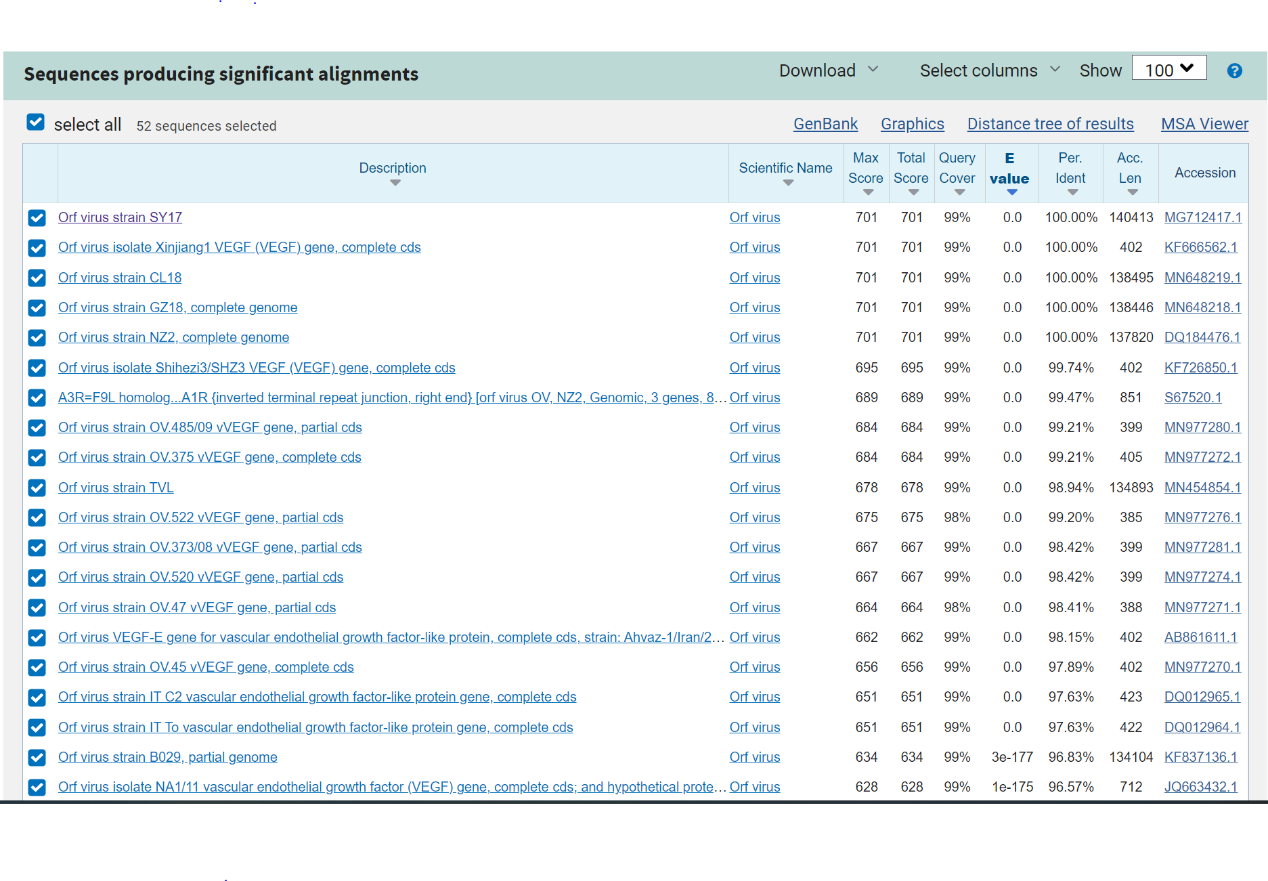


**vir sequence**

ATAAAAATTAGAAGCCTGATGCCGCAGTTGTTGATGAGGATGGTGAGTGCGCTGGTGCAGGCGGTGTGGCGCGCCAGCTTCTTGCTGGCCCCGTCGGCCACGGCAACGACCTTTCCGGATATCGTGATGGTGCAGGTGAAGCGCGGACAGTGATCCTCGCCGCCAGAACGCGTCTCGCAGAACTCCAGAGATCTGCGCGTCATCATGCAGAACTCGTTGACCGCGCTGACCGGGTTAAGACTTTTGAGGCGTATCACGGAAGACTGGGTCAGGATGTCGATGTCGCCTCCGAAAAGCGTATCGCACCCAGCCTCGGTCTCCATGGGCTCGGTGTCGGAGTTTTCGTCCTCCTCGGTGGGCGCGGCGGGCGCGCACTCTACGAACCAGCGGGGCGGGTTTCCGTCCTCGCAGCAAACGTCGTTCGAGTCCAGCAGGCGGTACAGCTGGCGGTTCGCCTCGTGTTTGGATATGCCGAGCTCCTTCGCGATCTGCTTGGCCGGCAGCTTGTCGCCGGATTTGCTGAGAAGCTCGAGGATCAGAGACGCG

**BLAST results:**


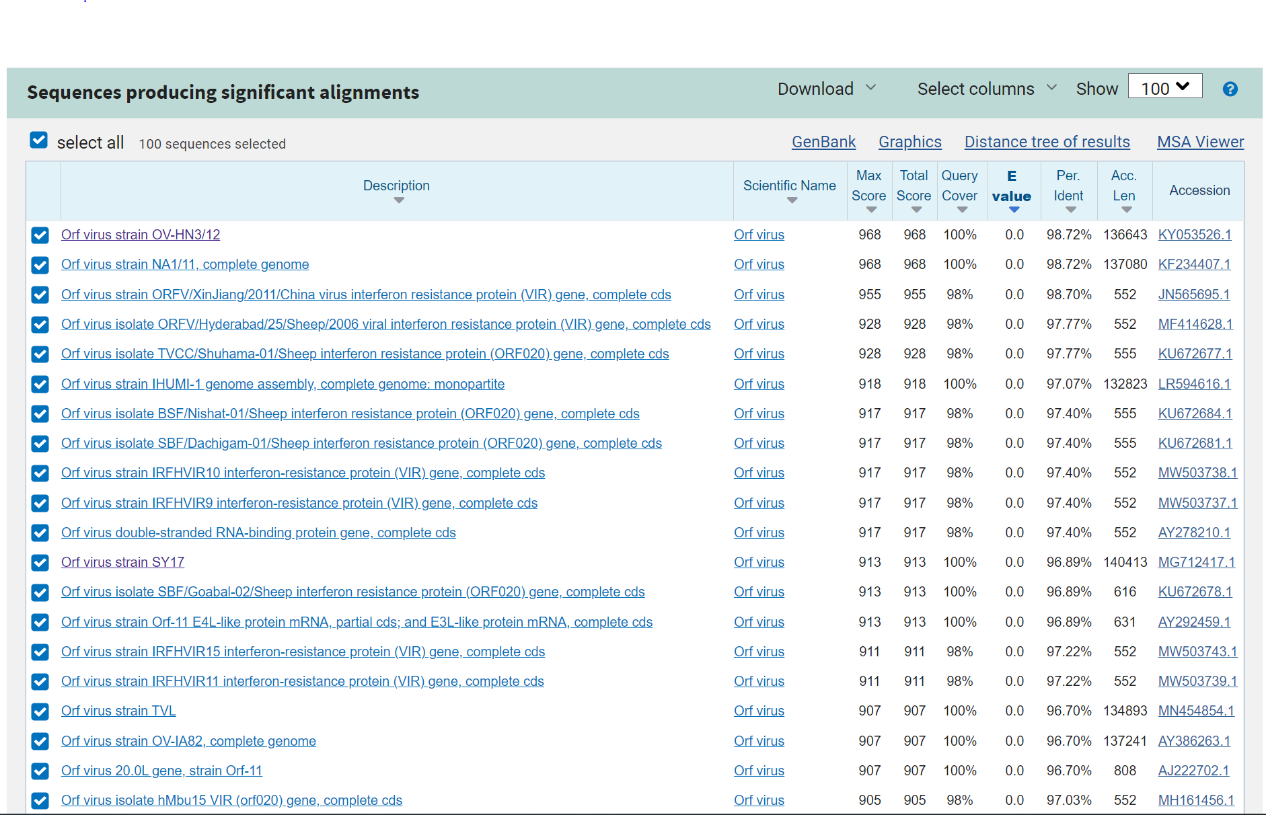


**2. The detailed clinical signs of each group were provided as following:**

| Group | Clinical signs post inoculation | Clinical signs post challenging |
| --- | --- | --- |
| rGS14-△CBP | Redness and swelling were observed on the 2^nd^ to 3^rd^ day after immunization and subsided on the 5^th^ to 7^th^ day. There was no difference between different groups. | There were 5 animals in the group, three of which presented orf related signs after challenging.  Clinical symptoms of diseased animals were as following: There were no obvious clinical signs at wound sites on the 1^st^ day after challenging and redness and swelling were observed on the 2^nd^ to 3^rd^ day. Ulcers were observed in the gingival wound on the 5^th^ to 7^th^ followed by the appearance of scabs outside the lip and granulomas in the gingival wound on the 9^th^ to 11^th^ day post challenging. 14 to 21 days after challenging, the crusts fell off and the wound at the gum gradually healed, forming white scar tissue.  And the clinical signs of uninfected animals were the same as the rGS14-△CBP-△GIF group. |
| rGS14-△GIF |  | There were 4 animals in the group, two of which presented orf related signs after challenging.  Clinical symptoms of diseased animals were as following: There were no obvious clinical signs at wound sites on the 1^st^ day after challenging and redness and swelling were observed on the 2^nd^ to 3^rd^ day. Ulcers were observed in the gingival wound on the 5^th^ to 7^th^ followed by the appearance of scabs outside the lip and granulomas in the gingival wound on the 9^th^ to 11^th^ day post challenging. 14 to 21 days after challenging, the crusts fell off and the wound at the gum gradually healed, forming white scar tissue.  And the clinical signs of uninfected animals were the same as the rGS14-△CBP-△GIF group. |
| rGS14-△CBP-△GIF |  | All the 4 animals in this group were not infected. And the clinical signs after challenging were as following: There were no obvious clinical signs on the 1^st^ day after challenging and redness and swelling were observed on the 3^rd^ to 5^th^ day followed by the subsidence of swelling from the 7^th^ to 11^th^ day, and there was a scar tissue in the inoculated site on the inner side of the lip. All the animals showed good state and normal feeding reaction. |
| PBS control |  | 4 animals in this group were all showed ORFV infected clinical signs after challenging.  The clinical signs were as following: There were no obvious clinical signs on the 1^st^ day after challenging. Severe redness and swelling reaction were observed on the 2^nd^ to 3^rd^ day after challenging followed by ruptured ulcer and festering on the 3^rd^ to 5^th^ day. Wound erosion was observed at the gum, which seriously affected feeding resulting in emaciation, rough and lusterless wool and increasing of body temperature. On the 11^th^ to 14^th^ day after challenging, scabs outside the lip were observed and the inflammatory reaction of the wound at the gum was relieved. And on the 14^th^ to 21^st^ day after challenging, the wound healed forming white scar tissue and the crusts gradually fell off. |
